# STYLE AND SUBSTANCE: A QUALITATIVE CHARACTERIZATION OF DEMENTIA CAREGIVING MANAGEMENT STYLES

**DOI:** 10.1093/geroni/igz038.2207

**Published:** 2019-11-08

**Authors:** Amanda N Leggett, Benjamin Bugajski, Breanna Webster, Brianna Broderick, Daphne Watkins, Laura Gitlin, Helen C Kales

**Affiliations:** 1 University of Michigan, Ann Arbor, Michigan, United States; 2 Drexel University, Philadelphia, Michigan, United States

## Abstract

Caring for a person living with dementia (PLWD) can take a physical and emotional toll, but understudied is the process of how family caregivers actually provide care (caregiver management styles). We interviewed 100 primary family caregivers regarding management of a recently experienced care challenge and values held which might impact care management decisions. Watkins’ (2017) rigorous and accelerated data reduction (RADaR) technique was used to analyze qualitative data through open/focused coding, determining commonalities of style components/themes, and finally defining caregiving management styles. Style for a given caregiver emerged from enacted care strategies, caregiver’s internal stances which informed their use of strategies, and broader engagement (or lack thereof) with the PLWD’s lived experience/reality. Styles emerging from the analysis will be described including the direct, rigid “Just do it” style, and the flexible, empathic “Teamwork” style. Individualizing caregiver interventions and supports based on caregiver management style is an important future direction.

